# Climate Change and Plant Foods: The Influence of Environmental Stressors on Plant Metabolites and Future Food Sources

**DOI:** 10.3390/foods14030416

**Published:** 2025-01-27

**Authors:** Ivana Šola, Danijela Poljuha, Ivana Pavičić, Ana Jurinjak Tušek, Dunja Šamec

**Affiliations:** 1 Department of Biology, Faculty of Science, University of Zagreb, Horvatovac 102a, 10000 Zagreb, Croatia; ivana.sola@biol.pmf.hr; 2 Department of Agriculture and Nutrition, Institute of Agriculture and Tourism, Karla Huguesa 8, 52440 Poreč, Croatia; danijela@iptpo.hr (D.P.); ipavicic@iptpo.hr (I.P.); 3 Faculty of Food Technology and Biotechnology, University of Zagreb, Pierottijeva 6, 10000 Zagreb, Croatia; ana.tusek.jurinjak@pbf.unizg.hr; 4 Department of Food Technology, University North, Trg Dr. Žarka Dolinara 1, 48000 Koprivnica, Croatia

**Keywords:** climate change, environmental stressors, phytochemicals, plant-based food sources

## Abstract

Climate change is reshaping global agriculture by altering temperature regimes and other environmental conditions, with profound implications for food security and agricultural productivity. This review examines how key environmental stressors—such as extreme temperatures, water scarcity, increased salinity, UV-B radiation, and elevated concentrations of ozone and CO_2_—impact the nutritional quality and bioactive compounds in plant-based foods. These stressors can modify the composition of essential nutrients, particularly phytochemicals, which directly affect the viability of specific crops in certain regions and subsequently influence human dietary patterns by shifting the availability of key food resources. To address these challenges, there is growing interest in resilient plant species, including those with natural tolerance to stress and genetically modified variants, as well as in alternative protein sources derived from plants. Additionally, unconventional food sources, such as invasive plant species and algae, are being explored as sustainable solutions for future nutrition.

## 1. Introduction

Plants have been fundamental to human existence for millennia, serving as a cornerstone of nutrition, health, and survival. They provide a wide range of essential nutrients vital for human development and overall health [1]. Beyond their nutritional value, plants are a rich source of bioactive compounds, including antioxidants and phytochemicals, which play crucial roles in preventing chronic diseases and promoting long-term health [2]. Moreover, plants hold cultural and culinary significance worldwide, while serving as primary producers in ecosystems. They are essential for maintaining biodiversity, regulating climate, and sustaining natural cycles such as carbon and water [1].

As the global population continues to grow and environmental challenges intensify, the urgency for sustainable food systems has never been greater. One promising strategy to address these challenges is the adoption of plant-based foods, which have the potential to significantly reduce the environmental footprint of food production while enhancing human health and nutrition [3]. However, plants, being sessile organisms, are particularly vulnerable to environmental changes. Unlike animals, they cannot move to escape adverse conditions, making them highly dependent on stable environmental factors such as temperature, water availability, soil quality, and sunlight. Consequently, their growth, development, and productivity are profoundly affected by shifts in these conditions. As climate change accelerates, the plants we rely on for food and raw materials today are likely to differ significantly in the future. Climate change poses unique challenges to agriculture, impacting crop yields, the availability of key plant species, and the production of critical nutritional and bioactive compounds [4].

Several factors will shape the future of food systems, with climate change being the most prominent. Over the past decade, the global climate has shifted significantly, and projections indicate continued and escalating changes. According to the Global Annual to Decadal Climate Update issued annually by the World Meteorological Organization (WMO), there is an 80 percent likelihood that the annual average global temperature will temporarily exceed 1.5 °C above pre-industrial levels within the next five years [5]. A second major factor is the adoption of strategies to mitigate climate change’s adverse effects. These strategies include advances in agricultural practices, food processing, and packaging technologies, such as the promotion of organic and ecologically sustainable approaches across the food supply chain [6]. For instance, the increasing incorporation of plant-based proteins in food processing provides a more sustainable alternative to traditional animal proteins [7]. Additionally, the integration of resilient crops—whether naturally stress-tolerant or genetically engineered—into agricultural systems offers a means of adapting to changing environmental conditions [8]. However, the success of these solutions will depend significantly on regulatory frameworks. While some policies may actively promote sustainable practices like organic farming, others may impose restrictions on the use of genetically modified (GM) crops, reflecting societal and political considerations. These interconnected factors—climate change, mitigation strategies, consumer behavior, and regulatory frameworks—will collectively determine the future of food systems. They will influence not only what crops are grown and consumed but also how food is produced, processed, and distributed sustainably.

This review explores how climate change is altering the nutritional composition and bioactive properties of plant food sources. It further examines the potential of diverse plant food sources, including resilient crop varieties, invasive species, and algae, to contribute to sustainable and nutritious food systems in the future.

## 2. Changes in Nutritional Value and Biological Activity of Plants Due to the Environmental Stresses

As we confront the realities of climate change, the landscape of food production, consumption, and nutritional value is rapidly evolving. Variations in temperature, precipitation, and atmospheric CO_2_ levels can affect the nutrient composition of plants. Climate-induced stressors can lead to significant changes in the phytochemical composition of plants. For example, higher temperatures may increase the levels of certain antioxidants while decreasing others, influencing the health benefits of these foods (reviewed by [9,10]). At the agricultural level, weeds can respond more favorably than crops to increased CO_2_ levels, which can affect crop production [4]. Higher CO_2_ concentrations might benefit the growth of invasive plants over native species, resulting in disruptions to ecosystem functions and biodiversity [11].

Changes in phenylpropanoid metabolism can be viewed as a general response of plant cells to various stress factors [12]. This can impact the nutritional value of plants in several ways, change the content of their antioxidants, alter flavor and aroma, and modify nutrient bioaccessibility (reviewed by [9,10]).

Herein, we summarize how various environmental stressors—such as extreme temperatures, water availability, UV-B radiation, and elevated levels of ozone and CO_2_, which are projected to intensify in the future—impact the nutritional composition and bioactivity of plant food sources. It is important to note that the results often vary significantly depending on the plant species, cultivar, growth stage, and experimental conditions. These factors may contribute to the contradictory findings frequently reported in the literature. Moreover, environmental stressors often occur simultaneously, such as the common combination of heat and drought, adding further complexity to experiments and data interpretation. Such multifactorial studies remain relatively scarce, highlighting the need for more integrated research approaches [13].

### 2.1. High Temperature

High temperature (HT), defined as a temperature exceeding the optimal range for the growth and development of a specific plant species or cultivar, influences plants during both the vegetative and reproductive stages. It affects the morphometric traits as well as phytochemical composition of plants. High temperatures adversely affect leaf function, flowering, seed development, and the composition of seeds [14]. HT can result in sunburn on leaves and twigs, promote leaf aging, hinder growth, create blotchy markings on fruits and leaves [15], and compromise grain quality [16]. The quality characteristics of aromatic rice are significantly affected by temperature, especially during the flowering and grain-filling stages [16]. Heat-stressed chickpea plants showed a significant reduction in yield-related traits, including pod count, seed count, and harvest index, with heat-sensitive genotypes being affected even more [17]. Examples of the changes in micro-, macronutrients, and bioactive compounds in different plant food sources are shown in Table 1.

As is evident from Table 1, HT in plants can reduce the levels of starch, protein, fat, and minerals, particularly affecting storage proteins, leading to lower seed weights and overall yield [16,18]. In mung beans, HT slowed seed filling and hindered starch and sucrose accumulation [18]. Similar reductions in total soluble proteins were observed in various plants, including chickpea [17], and maize [20] in which HT also disrupts carbohydrate metabolism, decreasing sugars and starch content. Not just the content, but the proportions of distinct protein fractions are also notably affected by HT [25]. For example, heat stress induces changes in the glutenin and gliadins ratio [25,26]. Increased temperatures impact wheat development by significantly reducing the grain-filling period, leading to changes in grain characteristics and protein accumulation [27]. During the grain-filling phase, HT can decrease the protein yield of white flour because more protein tends to bind to the aleurone layer, which is removed when producing white flour [18,28].

While HT increased glucosinolate accumulation in rocket salad [21], it may negatively impact consumer acceptance due to changes in flavor [29]. Increased glucosinolate content in rocket salad was positively correlated with perceptions of pepperiness, bitterness, and hotness [29]. HT also reduced chlorophyll, carotenoids, and oils in maize [20]. However, in kale and cabbage, chlorophyll *a* and *b* were increased by HT [23]. Among polyphenolic compounds, total phenolic acids and flavonoids in wheat were increased by HT [22], but total phenolics in kale and cabbage were decreased [23]. Aromatic metabolite 2-acetyl-1-pyrroline, the most important volatile aromatic compound in rice, was reduced under HT [16]. All this points out that HT may also change the aromas and influence the aroma and consumer acceptance of some plant food sources.

### 2.2. Low Temperature

Low temperatures (LT) are defined as a temperature below the optimal range for a given plant species or cultivar. Plants differ in their tolerance to chilling (0–15 °C) and freezing (<0 °C) temperatures, and resistance is species- or cultivar-dependent. In the literature, a common trend is that plants accumulate a variety of compatible solutes, including sugars, polyamines, glycine betaine, and proline, in response to LT and other osmotic stresses [30]. Examples of the changes in macronutrients, micronutrients, and bioactive compounds in different plant-based foods under low temperatures are shown in Table 2.

Cold-acclimated sweet cherry plants accumulated higher levels of total soluble sugars, reducing sugars, and sucrose compared to non-acclimated plants [31]. Similarly, palmate dragonhead herb produced a higher amount of total sugars under the effect of LT [37]. Also, broccoli microgreens grown under LT showed a 137% increase in soluble sugars and a 20% rise in total glucosinolates, although anthocyanins decreased by 81% [32]. Cold-water treatment further elevated total glucosinolates in broccoli [42]. LT also enhanced the soluble sugar levels in sugarcane roots, with higher increases in cold-tolerant varieties [33]. In walnut (*Juglans regia*) microclones, LT triggered a sevenfold rise in the juglone concentration, along with increases in flavones, phenolic acids, tannins, and terpenoids [34]. It also increased total phenolics and flavonoids in wheat sprouts [38]. Higher concentrations of total phenolic compounds, as well as individual polyphenolics such as caffeic acid, rosmarinic acid, cosmosiin, tilianin, luteolin glucosides, eriodictyol, naringenin, and apigenin, under the effect of LT were detected in palmate dragonhead herb [37]. LT effects on plant growth processes were variable. While LT delayed root development and mineral absorption in tomato seedlings [43], it improved the amino acid nutritional quality during rice flowering [41]. The carotenoid and flavonoid content decreased in kale sprouts under LT, whereas phenolic acids and total glucosinolates increased, with individual glucosinolates responding differently [36]. LT also induced cold-regulated and antifreeze proteins in freezing-tolerant wheat genotypes [44] and boosted soluble proteins in pepper varieties [35] and wheat grains [40]. Likewise, it enhanced the concentration of the minerals P, K, Ca, and Zn in wheat grains [39].

In terms of biological activity, LT-treated broccoli extracts showed enhanced anti-obesity (lipase inhibition) properties and increased α-amylase inhibitory activity compared to the controls in in vitro studies [32,42]. Cold stress in *Portulacaria afra* under LT in combination with water deficit decreased the antidiabetic potential through α-amylase inhibition [45]. Additionally, LT stress in pepper plants increased nonenzymatic antioxidants like ascorbate and glutathione [46], and in *Camellia japonica*, it boosted fatty acids like α-linolenic and jasmonic acid [47]. Fatty acids such as oleic acid, linoleic acid, linolenic acid, and total unsaturated acids were also increased by LT in palmate dragonhead herb [37]. However, LT reduced the potential of broccoli microgreen extracts to mitigate H_2_O_2_-induced damage in human and mouse cells [32]. It also diminished palmitic acid, arachidic acid, saturated acids, sesquiterpenes caryophyllene and germacrene, and flavonoid isorhoifolin in palmate dragonhead herb [37].

### 2.3. Drought

Drought is one of the most severe environmental stresses affecting plant productivity. Water makes up about 80–95% of the fresh biomass of a plant and plays a vital role in various physiological processes, including plant growth, development, and metabolism [48]. As a result, drought is often considered the primary environmental stress for many plants, particularly in drought-prone regions [49]. It is regarded as the single most critical threat to global food security in the future and has been a major catalyst for famines throughout history [50]. Drought significantly impacts various physiological, biochemical, and molecular processes within plants, leading to widespread changes in their growth, development, and survival and in the accumulation of nutrients and bioactive compounds in plant-based food (Table 3).

Mild drought enhanced flavonoids and phenolic acids in lettuce, improving its quality [51], while in young Chinese cabbage, total phenolic acids and proanthocyanidins increased, but total tannins decreased [52]. In durum wheat, total phenolics acids, and specifically ferulic acid, were increased by drought [61]. In licorice, soluble sugars and glycine betaine rose with prolonged drought [53], while water shortage in chicory boosted the ascorbic acid content [54]. Conversely, maize kernel oils were reduced under drought [20]. Glucosinolates increased in cabbage [55] and the glucoraphanin levels were elevated in pak choi [63], but total glucosinolates in savoy cabbage leaves [58] and brussels sprouts [60] were largely unaffected.

Photosynthetic pigments were also influenced. In maize hybrids, chlorophyll b and proteins decreased under drought, while the effects on chlorophyll a and carotenoids varied [20]. Conversely, drought enhanced the proteins related to synthesis in soybean [57]. Total amino acids were induced in savoy cabbage leaves [58].

Carbohydrate levels showed mixed responses: starch decreased in some maize hybrids [20], and in carrots, lutein increased under simulated drought, while lycopene and alpha-carotene decreased in certain cultivars but increased in others. Beta-carotene responses also varied by cultivar [59].

Drought stress enhanced essential oil composition in basil, increasing the levels of methylchavicol, methyleugenol, β-myrcene, and α-bergamotene, alongside upregulated biosynthesis gene expression [62].

### 2.4. Flood

Floods, unlike droughts, result in excessive water saturation of soils, severely impacting plant growth and productivity. Prolonged flooding leads to oxygen deprivation (hypoxia) in the root zone, disrupting respiration, nutrient uptake, and metabolic processes [64]. This can cause root decay, stunted growth, and reduced crop yields and affect the nutrient and bioactive compounds level (Table 4.)

Flooding stress caused varied responses in plants. Total tannins decreased in Chinese cabbage, while sugars increased [52]. In soybean, flooding influenced the synthesis of proteins and altered the nutrient levels, decreasing nitrogen and magnesium but increasing phosphorus, potassium, iron, manganese, zinc, and copper, with calcium and boron unaffected [70]. In sesame leaves [67], maize [20], and soybean [69], carotenoids were decreased by flooding.

Antioxidant activity showed mixed trends: flooding reduced the antioxidant capacity in sweet potato leaves but increased it in Chinese cabbage [52]. In rice seedlings, flooding reduced enzyme activity, chlorophyll synthesis, and overall productivity [65]. Chlorophylls were also reduced in sesame leaves [67,68] and soybean [69]. Wheat accumulated proline, sugars, soluble proteins, and free amino acids to counteract stress [66]. However, in cultivars with strong flood adaptation capabilities, metabolites may be minimally affected, as seen in the case of white cabbage [71].

In rice, superoxide dismutase activity increased under waterlogging, while catalase and ascorbate peroxidase activity decreased [72]. Similarly, barley responded to flooding with increased activity of superoxide dismutase, peroxidase, catalase, and ascorbate peroxidase, highlighting the need for high antioxidant activity and osmolyte accumulation to mitigate oxidative stress [73].

### 2.5. Increased Salinity

The Food and Agriculture Organization (FAO) of the United Nations has identified salinization as a critical global issue affecting agricultural production, food security, and sustainability, particularly in arid and semi-arid regions [74]. While the impact of soil salinization on crop production has long been recognized, its effects have intensified in recent years due to climate change. According to the FAO, climate change is accelerating salt accumulation in soils by driving the expansion of drylands, increasing water scarcity, and causing sea-level rise, which leads to saltwater intrusion in coastal regions [74]. This gradual buildup of salts in the soil often begins as a subtle issue but can escalate into severe land degradation if left unaddressed. Thus, increased salinity is a serious issue closely linked to climate change.

In a study on three Brassicaceae plants—Chinese cabbage, white cabbage, and kale—treatments with 50, 100, and 200 mM NaCl revealed varying outcomes [75]. Low to moderate salinity (50–100 mM) enhanced most phenolic compounds in the vegetables, whereas 200 mM led to a decline. Total glucosinolates increased in a dose-dependent manner, while total chlorophylls rose without significantly affecting the carotenoid content. Moreover, the macro- and micro-element balance remained largely stable under low to moderate salinity, with white cabbage showing increased calcium levels and minimal potassium loss. In a case of sweet pepper, increased salinity affects plants from the seed stage by reducing germination and later disrupts the balance of micro- and macronutrients in seedlings [76], potentially impacting the size and color of the fruits [77]. Similarly to the findings observed in Brassica plants, moderate salinity levels may also benefit the nutritional properties of sweet peppers [78]. Marra et al. [78] investigated the effects of varying sodium chloride concentrations (0, 50, and 75 mM) on bell pepper growth, nutrient content, and phytochemical profiles, aiming to establish an optimal salinity threshold. Their results showed that 75 mM NaCl not only avoided any detrimental impact on fruit quality but also produced significant improvements. Compared to the control group, this salinity level led to notable increases in total protein, total carbohydrates, lycopene, total carotenoids, and total phenols. Additionally, ascorbic acid levels and antioxidant activity were unaffected. Moderate salt stress demonstrated the most positive effects, enhancing the peppers’ nutritional value and concentrations of bioactive compounds such as minerals, phenolic acids, and flavonoids.

Increased salinity is a significant challenge tied to climate change, with its effects varying based on the concentration applied. The overall impact depends on the salt concentration, with moderate levels potentially enhancing the nutritional quality of food in some species. However, salinity is challenging to control in field-grown plants, and high soil salinity can have adverse effects. In such cases, strategies to mitigate these negative impacts should be implemented [79].

### 2.6. UV-B Light

Exposure to UV-B light has profound effects on plant secondary metabolism, enhancing both nutritional and phytochemical properties. In medicinal chrysanthemum, UV-B light increased the anthocyanin levels [80] and significantly boosted the contents of chlorophyll and carotenoids in the flowers. The activity of key enzymes in the phenylpropanoid pathway, such as phenylalanine ammonia-lyase (PAL) and 4-coumarate-CoA ligase (C4H), was also enhanced. Additionally, soluble sugars, amino acids, total flavonoids, vitamin C, and chlorogenic acid concentrations were all elevated in chrysanthemum flowers under UV-B exposure [80].

In lettuce (*Lactuca sativa* L.), UV-B radiation had no adverse effects on photosynthetic activity. Instead, it enhanced the overall antioxidant potential by increasing the levels of specialized metabolites, including total phenolics, flavonoids, anthocyanins, carotenoids, and ascorbic acid [81]. However, the effect of UV-B on lipid peroxidation varied between cultivars [81]. From a sensory perspective, UV-B radiation altered lettuce phytochemical composition, reducing sweetness while increasing bitterness. Furthermore, UV-B light specifically induced the production of ascorbic acid in lettuce [82,83].

In addition to UV-B, UV-A exposure also may enhance the antioxidant potential. For instance, lettuce exposed to UV-A showed higher 2,2-diphenyl-1-picrylhydrazyl (DPPH) radical-scavenging activity, indicating increased antioxidant capacity [84].

### 2.7. Ozone

Ozone treatment, a powerful abiotic stressor, has been shown to enhance the production of bioactive compounds and antioxidant activity in plants by activating key metabolic pathways. In *Melissa officinalis* L. shoot cultures, ozone induced the activity of phenolic metabolism enzymes, such as phenylalanine ammonia-lyase and cinnamyl alcohol dehydrogenase, resulting in a significant increase in the rosmarinic acid content. Extracts from ozone-treated *M. officinalis* shoots exhibited markedly higher antioxidant capacity, as measured by the DPPH method, compared to untreated plants [85]. Similarly, ozone exposure improved the phytochemical composition of *Rubus idaeus* raspberry fruit, elevating the phenolic compound levels and total antioxidant activity. Ozone treatment increased total phenolics, flavonoids, and vitamin C but reduced the anthocyanin content in raspberries [86]. In *Kalanchoe daigremontiana* cells, brief ozone exposure (10 ppm for 1 min) enhanced the levels of specific bioactive compounds, including total polyphenols, while improving the antioxidant capacity (measured by 2,2′-azino-bis(3-ethylbenzothiazoline-6-sulfonic acid) (ABTS) and DPPH assays) without causing phytotoxic effects. This treatment also significantly increased the activity of superoxide dismutase and catalase, enzymes crucial for mitigating oxidative stress by reducing reactive oxygen species (ROS) production [87].

### 2.8. CO_2_

Elevated CO_2_ (eCO_2_) enhances photosynthesis by reducing the oxygenation reaction of Rubisco, increasing sugar accumulation and respiratory intermediates like organic acids, which influence the plant growth, antioxidant properties, and phytochemical composition in C3 plants [88]. In fennel, eCO_2_ reduced antioxidant activity but increased the flavonoid levels [89], while in oil palm, it boosted total phenolics, flavonoids, soluble sugars, starch, and non-structural carbohydrates, while reducing proteins and leaf nitrogen [90]. Parsley and dill grown under eCO_2_ showed elevated levels of phenolics, flavonoids, vitamins A and E, organic acids, amino acids, and antioxidant capacity, alongside enhanced antiprotozoal, antibacterial, and anticancer properties [91]. Similarly, caraway exhibited improved phytochemical and antioxidant activity, with sprouts showing stronger effects than mature plants, and antibacterial activity increasing at the mature stage [92]. In broccoli, eCO_2_ stimulated glucosinolate accumulation (e.g., glucoraphanin), myrosinase activity, and sulforaphane production, enhancing the anticancer and anti-inflammatory properties by inhibiting COX-2 and lipoxygenase, while boosting glutathione-S-transferase and quinone reductase activity [93]. eCO_2_ also increased the therapeutic compounds in *Hymenocallis littoralis* bulbs, improving the anticancer and antiviral properties [94], and in *Portulacaria afra* leaves, it raised the flavonoid levels and antimicrobial activity [95]. In tomato seedlings, eCO_2_ improved photosynthesis, biomass, and antioxidant enzyme activities, alleviating oxidative stress [59]. However, adverse effects were noted in soybean, where eCO_2_ reduced key antioxidant enzymes, Rubisco activity, chlorophyll, and carotenoid levels, suggesting a decline in antioxidant defenses under prolonged eCO_2_ exposure [96]. Overall, elevated CO_2_ promotes phytochemical enrichment, antioxidant potential, and therapeutic properties in many plants, though the effects vary by species, growth stage, and metabolic pathways.

## 3. Future Food Sources

### 3.1. Plant Protein Sources

**Figure 1:**
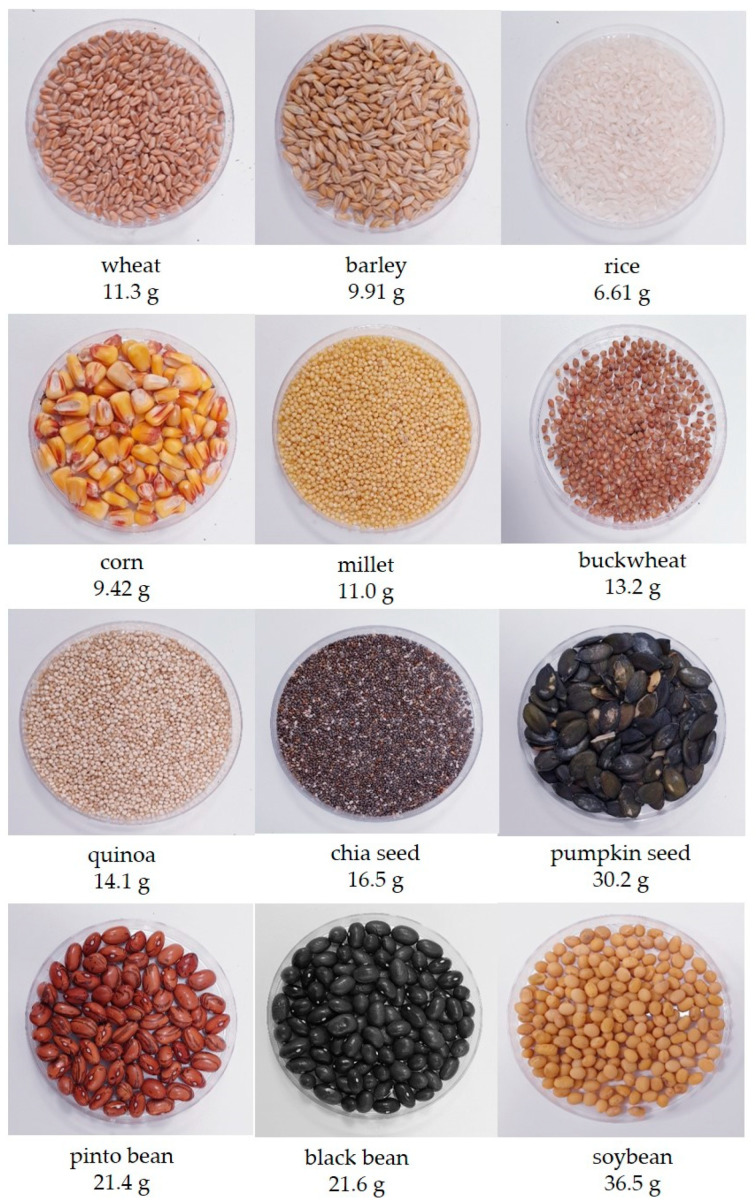
Examples of plant protein source with the protein content in 100 g according to FoodData Central [105].

In the future, high-protein plants are expected to play a pivotal role in addressing global food security, sustainable agriculture, and environmental challenges [97]. The interest in plant-based proteins has surged due to their applications in both the food and non-food industries, as well as their environmentally friendly, biodegradable nature [98]. This topic has been extensively researched and reviewed in recent years [99,100,101,102]. Plants are a promising source of proteins for diverse uses, offering advantages such as lower production costs, abundant protein yields, and relatively simple and efficient extraction methods [103]. For centuries, agricultural crops have served as key food sources, providing high amounts of protein, starch, and oil. Major sources of plant proteins include cereal grains, legumes, pulses, nuts, and seeds from various fruits and vegetables. These sources are well suited for direct use in producing protein-based bioproducts [104]. Examples of plant protein sources and their protein content per 100 g of raw material, as reported by FoodData Central [105], are illustrated in Figure 1.

In 2019, 1.16 billion tons of cereal were utilized for food worldwide [106]. Cereals are one of the main foodstuffs consumed around the world. Cereals are plants of the Poaceae family that are grown for their edible constituents. Wheat (*Triticum* sp.), rice (*Oryza* sp.), and maize (*Zea mays* L.) are the major cereal grains. Other representatives are barley (*Hordeum vulgare* L.), rye (*Secale cereal* L.), oats (*Avena sativa* L.), sorghum (*Sorghum* sp.), millets (*Cenchrus* sp.), etc. Edible grains from other plants that do not belong to the Poaceae family but are used as grains are known as pseudocereals such as amaranth (Amaranthus sp.), quinoa (*Chenopodium quinoa* L.), and buckwheat (*Fagopyrum esculentum* L.) [107]. A major source of dietary energy in cereal grains is starch while the protein content ranges from 8 to 15% *w*/*w* [108]. The storage proteins are most abundant in the seed. Whole cereal grains represent a good source of fibers, vitamins, and minerals [109].

Legumes and pulses are another important plant protein source. Pulses are dry seeds of leguminous plants with a low fat content compared to leguminous oil seeds [110]. Soybeans (*Glycine max* L) are currently the most commonly used crop for protein production. Legumes also comprise chickpeas (*Cicer arietinum* L.), peas (*Pisum sativum* L.), different varieties of beans (*Phaseolus vulgaris* L.), lentils (*Lens culinaris* Medik), lupin bean (*Lupinus* sp.), etc. Legumes and pulses are an important part of the human diet. Beans, peas, and lentils contain less than 30% *w*/*w* of protein content while soybeans and lupin can contain approximately 40% *w*/*w* of protein [111]. Beyond dietary fibers, vitamins, and minerals, legumes and pulses are also sources of fat and carbohydrates [104].

Seeds of almond (*Prunus amygdalus* L.), walnut (*Juglans regia* L.), cashew (*Anacardium occidentale* L.), pistachio (*Pistacia vera* L.), pine nut (*Pinus* sp.), hazelnut (*Corylus avellana* L.), sesame (*Sesamum indicum* L.), and pumpkin (*Cucurbita* sp.) belong to the nuts and seeds category. Although some nuts and seeds are allergenic, they are important plant-based proteins and represent valuable sources of lipids, fibers, minerals, and vitamins [112]. Nuts and seeds contain 18–20% and 20–40% *w*/*w* of protein content, respectively. It is important to note that these protein sources are not commonly used for commercial protein extractions, they are mainly used for edible oil extraction [110]. By-products of oil production (meals, skins, hulls), although used as animal feed, have potential for applications as protein sources [113].

Advances in biotechnology and precision farming could further enhance protein yield and quality, enabling the efficient use of these plants in creating sustainable, plant-based alternatives to animal-derived products, benefiting both human health and the planet [114]. However, a full substitution may face challenges such as meeting the specific amino acid profiles of animal proteins, cultural and dietary preferences, and scalability of plant-based production systems [115]. Innovations like blending plant proteins with cultured (lab-grown) meat or fermentation-derived proteins could also bridge the gap [116]. While a complete replacement of meat proteins by plant-based proteins may not happen universally, they are likely to coexist and play an increasingly significant role in reducing the reliance on traditional animal farming.

### 3.2. Resilient Crops

Some approaches to mitigating the negative impacts of climate change on crops and plant-based foods include the increased use of naturally resilient plants or the creation of resilient plants through breeding and genetic engineering [8]. Naturally resilient crops are plant species that have evolved to withstand harsh conditions such as drought, salinity, pests, and extreme temperatures [117]. Examples include sorghum, millet, and teff, which thrive in arid environments, and salt-tolerant plants like mangroves and saltbush. These crops provide a foundation for future breeding programs aimed at enhancing resilience in modern agriculture [117]. By harnessing their natural traits through traditional breeding or advanced biotechnology, such as CRISPR gene editing, scientists can develop high-yielding crops that are better adapted to climate change and resource-limited settings [8]. However, the adoption of naturally resilient crops or even more genetically modified crops faces challenges, as there are concerns that consumers may be hesitant to accept such foods [118]. A potential solution is to promote these crops as healthier options. A good example is the promotion of kale (*B. oleracea* var. *acephala*) in the U.S., where its consumption has steadily increased over the last decade [119]. Kale is naturally more resistant to environmental stress than other *Brassica* species, making it easier to grow under unfavorable climate conditions [120]. According to the literature, kale contains a comparable amount of bioactive or “health” compounds to other Brassica plants, but its superior environmental adaptability makes it a more practical choice for cultivation [119]. This ease and cost-effectiveness of growing kale, coupled with its resilience, are likely reasons for its widespread promotion as a superfood and consequently its increased production and consumption [119]. While kale is often considered a superfood, current research does not conclusively show that it provides more health benefits than other Brassica vegetables, which all generally contain bioactive compounds [119]. However, further studies could shed light on whether its nutritional and health advantages are more significant.

The acceptance of genetically modified (GM) plants that are more resilient to climate change remains a complex and often polarizing topic. On one hand, these crops offer significant potential to address the challenges of global food security by thriving in adverse conditions such as drought, extreme temperatures, salinity, and pest outbreaks [8]. Examples include drought-tolerant maize and salt-resistant rice, which have been developed to ensure stable yields under environmental stresses intensified by climate change [8]. However, public acceptance of GM plants varies widely across regions and is often shaped by cultural, regulatory, and economic factors [118]. In countries like the United States, GM crops are widely adopted and accepted in agriculture, particularly for staple crops like corn and soybeans [118]. In contrast, European countries exhibit higher levels of skepticism, largely due to concerns about potential health risks, environmental impacts, and corporate control over seed supply. The future use of resilient plants will largely depend on regional regulations and public attitudes toward GM food. In the United States, it is likely that more GM resilient plants will be adopted to address the challenges posed by climate change. In contrast, Europe, with its stricter regulations and greater consumer skepticism toward GM foods, may focus on naturally resilient plants. This approach may require significant adaptations in farming systems and a shift in consumer habits to embrace these naturally resilient crops or new food sources.

### 3.3. New Food Sources

#### 3.3.1. Invasive Plant Species as a Nutritional Resource

**Figure 2:**
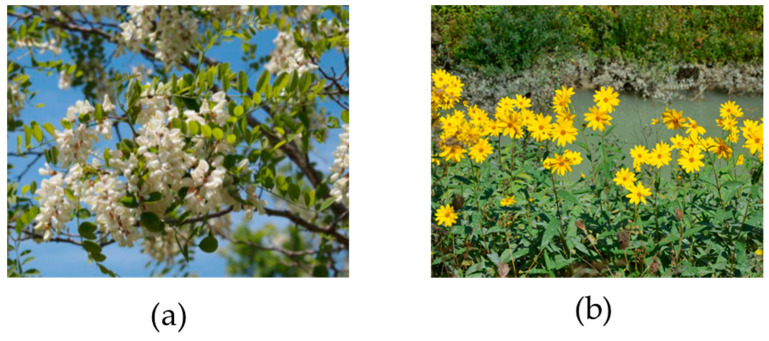
Widespread invasive plant species with dietary value for human consumption. (**a**) Black locust (*Robinia pesudoacacia* L.); (**b**) Jerusalem artichoke (*Helianthus tuberosus* L.).

Invasive plant species, often regarded as ecological threats, are increasingly recognized for their potential as food sources due to their rapid growth and resilience in various climates. These plants are nutrient-rich, containing essential vitamins, minerals, and antioxidants that could contribute to human health. For example, Japanese knotweed (*Reynoutria japonica*) is rich in resveratrol, an antioxidant with anti-inflammatory properties [121], while kudzu (*Pueraria montana var. lobata*), due to its high medicinal and nutritional value, known as the ‘Asian ginseng’, contains beneficial isoflavones [122]. Edible flowers of invasive black locust (*Robinia pseudoacacia* L.) (Figure 2a) have also been identified as high-value food additives due to their biological benefits [123,124,125,126]. Jerusalem artichoke (*Helianthus tuberosus*) (Figure 2b) is another versatile and nutrient-rich invasive plant with applications as a functional food and bioactive ingredient, offering benefits such as lowering blood sugar, cholesterol, and triglycerides, promoting weight loss, improving metabolism, and supporting gut health [127]. Additionally, its leaf protein concentrate provides a sustainable alternative protein source with high yields (averaging 33.3% dry mass) and bioactive phytochemicals, underscoring its value for human and animal nutrition [128]. Proper processing methods, such as thermal and enzymatic treatments, are necessary to mitigate anti-nutritional factors like oxalates and tannins. These methods enhance nutrient bioavailability and reduce toxicity [129].

However, there is a limited number of studies on the human consumption of invasive plants [130,131,132,133]. While harvesting these species for human consumption could contribute to biodiversity conservation by reducing their populations, it might also inadvertently encourage their persistence or spread, worsening the ecological impacts. Transforming invasive plants into valuable resources could foster cultural acceptance, complicating their management and control [134]. Despite these challenges, this approach offers a sustainable food source and an opportunity to raise public awareness about invasive species and their environmental effects.

#### 3.3.2. Algae: A Climate-Resilient Food

Algae, including microalgae like spirulina and chlorella, and macroalgae such as kelp and nori, are sustainable and versatile food sources [135]. They are rich in proteins, omega-3 fatty acids, vitamins, and minerals and contain bioactive compounds like carotenoids, polyphenols, and phycocyanins, which offer antioxidant, anti-inflammatory, and antimicrobial properties [136]. *Chlorella vulgaris*, for instance, contains proteins (43–61%), essential amino acids, and significant non-essential amino acids, making it a high-quality protein source [137]. It also has vitamins, minerals, and omega-3 fatty acids, offering a beneficial alternative to animal proteins [137].

*C. vulgaris* proteins, though nutritious, are limited by a rigid cellulose cell wall that reduces digestibility. Pre-treatment methods like bead milling and sonication enhance nutrient accessibility [136,138,139]. Despite its benefits, *C. vulgaris* may accumulate toxic heavy metals like cadmium and lead, raising safety concerns [140]. Future research could explore its use in functional foods for specific health outcomes, such as immune support or diabetes management.

For macroalgae like kelp and nori, chemical characterization reveals bioactive compounds like polysaccharides, omega-3 fatty acids, antioxidants, and minerals (iodine, selenium, iron) that contribute to health benefits, including antioxidant, anti-inflammatory, immune-boosting, and cholesterol-lowering effects [141]. Kelp (*Saccharina japonica*), a brown seaweed, is rich in minerals but may also contain harmful elements like iodine and arsenic, which require careful monitoring [142,143]. Processing methods such as steaming and boiling reduce the iodine and arsenic content [144]. Nori (*Porphyra umbilicalis*), a red macroalga, is a rich source of vitamin B12, beneficial for vegan diets, and its polysaccharides act as prebiotics, supporting gut health [145].

Recent advancements in spectroscopy (Fourier Transform Infrared Spectroscopy, Near-Infrared Spectroscopy, and/or Raman Spectroscopy) and chromatography and the coupling of chromatography with spectroscopy [146] enhance the detection of bioactive and toxic compounds in algae, ensuring the safety and efficacy of supplements and functional foods. As part of global food security strategies, algae are increasingly recognized as valuable, sustainable food sources [135].

Microalgae can be incorporated into the human diet in various forms. The simplest method is direct consumption of dried microalgal biomass, such as Spirulina or Chlorella, which can be consumed as tablets, powder, or flakes [147]. These can be added to smoothies, juices, or sprinkled on salads and soups. Microalgae can also be used as ingredients in a wide range of food products. They can be incorporated into baked goods like bread, cakes, and crackers, adding nutritional value and a unique flavor [148]. Microalgae extracts can be used as natural colorants and flavorings in foods and beverages [149]. For example, blue-green algae can provide a vibrant blue color, while other species can impart earthy or nutty flavors. Furthermore, microalgae can be used to enrich the nutritional content of other foods. For instance, they can be added to animal feed, which can then transfer the beneficial nutrients to meat, eggs, and dairy products [150]. This approach can enhance the nutritional quality of animal-based foods while reducing the environmental impact of livestock production. As research and technology advance, new and innovative ways of incorporating microalgae into the human diet are constantly being explored. These include the development of microalgae-based protein isolates, which can be used as ingredients in plant-based meat alternatives, and the use of microalgae to produce single-cell protein, a high-protein food source that can be used in various applications.

## 4. Conclusions and Further Perspective

Climate change, characterized by shifts in temperature, precipitation patterns, and other environmental factors, is significantly impacting agricultural production and food security. This review summarizes the current data on how environmental stressors—including extreme temperatures, water scarcity or excess, increased salinity, elevated UV-B radiation, and increased atmospheric ozone and CO_2_—affect the nutritional composition and bioactivity of plants. These changes influence macro-elements and phytochemicals, altering the suitability of specific crops and driving shifts in agricultural practices and dietary habits. The extent of these changes is strongly influenced by the intensity of specific stressors, the type of crop, and various other factors. As a result, under certain conditions, metabolite levels may increase, while under others, they may decrease. Although research in this area has expanded in recent years, data remain limited. Most studies have focused on a narrow range of metabolites, using primarily targeted metabolomic analysis. However, with advancements in modern metabolomic techniques, more comprehensive data are expected to emerge in the future. Additionally, the impact of these environmental changes on the bioactivity of plant-based food sources and, indirectly, human health, remains unclear. As we have highlighted, the majority of available data come from in vitro studies, and gaps remain, especially regarding the influence of various stressors. Therefore, future research should prioritize understanding how climate-induced changes affect the bioactivity of food, ideally through animal or human models.

Resilient plants, including naturally tolerant species and genetically modified varieties, are emerging as vital solutions to sustain global food systems under changing climatic conditions. In addition, alternative protein sources, such as invasive plant species and algae, offer promising options. However, introducing new foods into diets or altering existing dietary patterns is a complex process, requiring time and the consideration of various social and ethical issues. The multifaceted impacts of climate change on plant food sources call for a comprehensive, interdisciplinary approach. Studies should involve collaboration among researchers from agriculture, nutrition, environmental science, and social sciences. Such interdisciplinary efforts will be essential for developing sustainable solutions for future food systems, addressing both the scientific and societal challenges posed by climate change.

## Figures and Tables

**Table 1:** Examples of the changes in macronutrients, micronutrients, and bioactive compounds of plant food sources under heat stress.

	Plant Food Sources	Plant Part	Temperature (Day/Night)	Impact of High Temperature	Reference
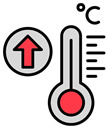 High temperature	mung bean (*Vigna radiata*)	leaves	42 °C/30 °C	↓ starch, protein, fat, minerals, storage proteins	[18]
	broccoli (*Brassica oleracea* L. convar. *botrytis* (L.) Alef. var.*cymosa* Duch.)	sprouts	38 °C/33 °C	↑ As, Co, Cr, Hg, K, Na, Ni, Pb, Se, and Sn ↓ Ca, Cd, Cu, Mg, Mn, P	[19]
	chickpea (*Cicer arietinum*)	leaves	32 °C/20 °C	↓ Ca, P, and Fe ↓ total souluble proteins ↓ starch	[17]
	maize (*Zea mays*)	leaves	≤40 °C (data day/night not available)	↓ total soluble proteins ↓ starch ↓ chlorophyll *a* and *b*, carotenoids	[20]
	rocket salad (*Diplotaxis tenuifolia* and *Eruca sativa*)	leaves	40 °C/30 °C	↑ glucosinolate	[21]
	wheat (*Triticum* spp.)	grains	30 °C (data day/night not available)	↑ total phenolic acids, total flavonoids, palmitic acid, oleic acid ↓ linoleic acid, linolenic acid, campesterol	[22]
	kale and cabbage (*Brassica oleracea*)	leaves	32 °C (data day/night not separated)	↑ chlorophylls ↓ total phenolics	[23]
	potato (*Solanum tuberosum*)	tubers	increase from optimal temperature for 1 °C, 2 °C, 3 °C	↓ Fe, Cu, Zn	[24]

↓ represents decrease; ↑ represents increase.

**Table 2:** Examples of the changes in macronutrients, micronutrients, and bioactive compounds of plant food sources under low-temperature stress.

	Plant Food Sources	Plant Part	Temperature	Impact of Low Temperature	Reference
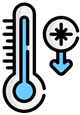 Low temperature	sweet cherry (*Prunus avium*)	bark of shoots	−25 °C (data day/night not separated)	↑ total soluble sugars, reducing sugars, and sucrose	[31]
	broccoli (*Brassica oleracea* L. convar. *botrytis* (L.) Alef. var. *cymosa* Duch.)	microgreens	12 °C/7 °C	↑ total soluble sugars, glucosinolates ↓ anthocyanins	[32]
	sugarcane seedlings (*Saccharum officinarum*)	seedlings	4 °C (data day/night not separated)	↑ total soluble sugar	[33]
	walnut (*Juglans regia*)	shoots	3–5 °C (data day/night not separated)	↑ juglone, flavones, phenolic acids, tannins, polysacharids, alkaloids, fatty acid esters, terpenoids, along with other volatile components	[34]
	pepper (*Capsicum annuum*)	seedlings	4 °C (data day/night not separated)	↑ soluble proteins	[35]
	flat leaf kale (*Brassica oleracea* var. *acephala*)	leaves	8 °C (data day/night not separated)	↑ chlorophylls, phenolic acids, flavonoids, carotenoids, glucosinolates	[33,36]
	palmate dragonhead herb (*Dracocephalum palmatum*)	leaves	1 °C (data day/night not separated)	↓ palmitic acid, arachidic acid, saturated acids, caryophyllene, germacrene, isorhoifolin ↑ oleic acid, linoleic acid, linolenic acid, unsaturated acids, caffeic acid, rosmarinic acid, cosmosiin, tilianin, luteolin glucosides, eriodictyol, naringenin, apigenin, total phenolic compounds, total sugars ≈ total fat acids, total proteins	[37]
	wheat (*Triticum aestivum*)	sprouts	<4 °C (data day/night not separated)	↑ total phenolics, total flavonoids	[38]
	wheat (*Triticum aestivum*)	grains	0 °C/10 °C −2 °C/8 °C −4 °C/6 °C	↑ P, K, Ca, Zn, proteins ↓ starch	[39,40]
	rice (*Oryza sativa*)	grains	6 °C/12 °C	↑ amino acids	[41]

↓ represents decrease; ↑ represents increase; ≈ not influenced.

**Table 3:** Examples of the changes in macronutrients, micronutrients, and bioactive compounds of plant food sources under drought.

	Plant Food Sources	Plant Part	Impact of Drought	Reference
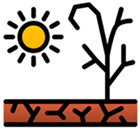 Drought	lettuce (*Lactuca sativa*)	leaves	↑ polyphenols, flavonoids	[51]
	Chinese cabbage (*Brassica rapa* ssp. *pekinensis*)	leaves	↑ phenolic acids, proanthocyanidins, total sugars ↓ total tannins	[52]
	licorice (*Glycyrrhiza glabra*)	leaves and root	↑ soluble sugars, glycine betain	[53]
	common chicory (*Cichorium intybus*)	leaves	↑ ascorbic acid	[54]
	maize (*Zea mays*)	kernel	↓ oil, chlorophylls, proteins, starch	[20]
	cabbage (*Brassica oleracea* L. *capitata* Group)	head tissues	↑ glucosinolate, proline, phenolic acids, flavonoids, total polyphenols, ↓ total chlorophylls ≈ carotenoids	[55,56]
	soybean (*Glycine max* L. cultivar Enrei)	root	↑ proteins	[57]
	savoy cabbage (*Brassica oleracea* convar. *capitata* var. *sabauda*) [58]	leaves	↑ total aminoacids ≈ glucosinolates	[58]
	carrot (*Daucus carota*)	taproots	↑ lutein ↓ lycopene	[59]
	Brussels sprouts (*Brassica oleracea*)	leaves	≈ glucosinolates	[60]
	durum wheat (*Triticum turgidum* ssp. *durum*) [61]	grains	↑ ferulic acid, total phenolic acids	[56,61]
	basil (*Ocimum basilicum*)	essential oil from leaves	↑ methylchavicol, methyleugenol, b-myrcene and a-bergamotene in basil essential oil	[57,62]

↓ represents decrease; ↑ represents increase; ≈ not influenced.

**Table 4:** Examples of the changes in macronutrients, micronutrients, and bioactive compounds of plant food sources under flood.

	Plant Food Sources	Plant Part	Impact of Flood	Reference
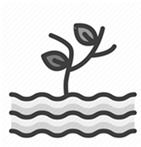 Flood	Chinese cabbage (*Brassica rapa* ssp. *pekinensis*)	leaves	↑ sugars	[52]
	rice (*Oryza sativa*)	seedlings	↓ chlorophylls	[65]
	wheat (*Triticum aestivum*)	leaves	↑ proline, sugars, soluble proteins, and free amino acids	[66]
	sesame (*Sesamum indicum*)	leaves	↓ chlorophylls and carotenoids, ascorbic acid	[67,68]
	maize (*Zea mays*)	kernel	↓ carotenoids	[20]
	soybean (*Glycine max*)	leaves	↓ chlorophylls, proteins, N, Mg, carotenoids ↑ P, K, Fe, Mg, Zn, Cu, Ca, B	[69,70]

↓ represents decrease; ↑ represents increase; ≈ not influenced.

## Data Availability

No new data were created or analyzed in this study. Data sharing is not applicable to this article.
